# Syncytin 1, CD9, and CD47 regulating cell fusion to form PGCCs associated with cAMP/PKA and JNK signaling pathway

**DOI:** 10.1002/cam4.2173

**Published:** 2019-04-25

**Authors:** Fei Fei, Chunyuan Li, Xinlu Wang, Jiaxing Du, Kai Liu, Bo Li, Peiyu Yao, Yuwei Li, Shiwu Zhang

**Affiliations:** ^1^ School of Medicine Nankai University Tianjin P.R. China; ^2^ Department of Pathology Tianjin Union Medical Center Tianjin P.R. China; ^3^ Graduate School Tianjin University of Traditional Chinese Medicine Tianjin P.R. China; ^4^ Tianjin Medical University Tianjin P.R. China; ^5^ Department of colorectal surgery Tianjin Union Medical Center Tianjin P.R. China

**Keywords:** cell fusion, colorectal cancer, polyploid giant cancer cells, syncytin 1

## Abstract

**Background:**

We have previously reported the formation of polyploid giant cancer cells (PGCCs) through endoreduplication or cell fusion after cobalt chloride (CoCl_2_) induction. Cell fusion plays an important role in development and disease. However, the underlying molecular mechanism concerning cell fusion in PGCCs formation and clinicopathological significances remains unclear.

**Methods:**

We treat HCT116 and LoVo cell with CoCl_2_ and observed the cell fusion via fluorescent markers of different colors. Western blot and immunocytochemical staining were used to compare the expression and subcellular location of the fusion‐related proteins syncytin 1, CD9, and CD47 along with PKA RIα, JNK1, and c‐Jun between PGCCs and control cells from the HCT116 and LoVo cell lines. Moreover, 173 cases of colorectal tumor tissue samples were analyzed, including 47 cases of well‐differentiated primary colorectal cancer (group I) and 5 cases of corresponding metastatic tumors (group II), 38 cases of moderately differentiated primary colorectal cancer (group III) and 14 cases of corresponding metastatic tumors (group IV), and 42 cases of poorly differentiated primary colorectal cancer (group V) and 27 cases of corresponding metastatic tumors (group VI).

**Results:**

The expression of syncytin 1, CD9, and CD47 is higher in PGCCs than in control cells and they are located in the cytoplasm. The expression of PKA RIα and JNK1 decreased, and that of c‐Jun increased in PGCCs. The syncytin 1 expression was significantly different between groups I and II (*P* = 0.000), groups III and IV (*P* = 0.000), groups V and VI (*P* = 0.029), groups I and III (*P* = 0.001), groups III and V (*P* = 0.000), and groups I, III, and V (*P* = 0.000).

**Conclusions:**

These data indicate that the cell fusion‐related proteins syncytin 1, CD9, and CD47 may be involved in PGCC formation, and that cAMP/PKA and JNK signaling is likely to promote PGCC formation via the regulation of cell fusion processes.

## 1 INTRODUCTION

Polyploid giant cancer cells (PGCCs) refer to a special subpopulation of cancer cells that were previously considered to be senescent cells without dividing ability or believed to be at the stage of mitotic catastrophe. Recent studies have confirmed that PGCCs possess properties of cancer stem cells, with the expression of the cancer stem cell markers CD44 and CD133, and therefore, promote tumor maintenance and recurrence. In addition, these cells were also identified to differentiate into benign tissues including adipose, cartilage, and bone tissues. We previously demonstrated that the number of PGCCs increased along with the malignant grade of the tumor, and that the daughter cells generated by PGCCs via budding acquired a mesenchymal phenotype and displayed stronger capacities of migration and invasion than control cells. PGCCs differ markedly from diploid cancer cells in morphology, size, tumorigenicity, radioresistance, and chemoresistance. Furthermore, we previously showed that PGCCs were able to generate erythrocytes expressing embryonic and fetal hemoglobin with a high O_2_‐binding affinity, satisfying the transitional need of tumor cells in the hypoxic condition. Surprisingly, PGCCs or other cancer cells and these erythrocytes contribute to the formation of vasculogenic mimicry (VM), which might promote the formation of a complementary blood supply network to support the growth, invasion, and metastasis of cancer cells. It has recently been recognized that PGCCs contribute to the heterogeneity of solid tumors and that they are the most commonly described histological features in the pathologic diagnosis of tumors, with significant variation in nucleus shape and size. Given that these features and functions of PGCCs have been shown, no specific conclusion for the mechanism of PGCC formation has yet been reached. We speculate that the CoCl_2_‐induced formation of PGCCs is related mainly to endoreduplication or cell fusion. Recent findings have made great progress in detecting the role of cell cycle‐related proteins including Cyclin B1, CDC25, and Cyclin E in PGCC formation. However, how the cell fusion process functions in PGCC formation is not yet clear.

Cell fusion is a vital and highly regulated event in development and tissue homeostasis.^1^ Spontaneous cells fusion or syncytialization is common in neoplasia and viral infections; however, it is relatively restricted in healthy tissues.^2^ Cell fusion has been confirmed to be involved in processes as sperm‐oocyte fusion, the formation of osteoclasts and muscle fibers, the development of the placenta, and in giant cell formation during chronic inflammatory reactions.^3^, ^4^, ^5^ Cell‐cell fusion has previously been reported in cancer,^6^ which might contribute to the formation of aneuploidy and alter biological behavior.^7^ Advances in some studies speculate that cell fusion is one of the driving factors of tumor initiation and progression. Therefore, it is reasonable to postulate that the formation of PGCCs is highly correlated with cell fusion. Although the molecular mechanisms underlying cell‐cell fusion events are not yet well understood, several known promising candidates for fusion‐related proteins are the members of the syncytin family of proteins, CD9 and CD47.^7^, ^8^, ^9^ Syncytin 1 is encoded by endogenous retrovirus family W, env(C7), member 1 (ERVWE1) and is the first identified fusogenic protein functionally involved in the regulation of placental cell fusion through an interaction with its ubiquitously expressed receptor, solute carrier family 1, members 4 and 5 (SLC1A4 and SLC1A5).^10^, ^11^ Syncytin 1 is involved in the formation of the syncytiotrophoblast and has been implicated in neoplastic cell fusion.^2^, ^12^ CD9, belonging to the tetraspan membrane protein family, has been shown to be implicated in diverse functions involving cell signaling, growth, motility, and metastasis. Moreover, as previously reported, CD9 expression plays a crucial role in cell adhesion and morphological changes, and in sperm‐egg fusion.^13^ CD47 is a transmembrane glycoprotein belonging to the superfamily of immunoglobulins. The recent finding that CD47 acts as one of the regulatory factors for the fusion of macrophages and the cell fusion‐induced osteoclast formation led us to realize the importance of CD47 in cell fusion events.^14^, ^15^ Hence, we measured the expression level of the fusion‐related proteins syncytin 1, CD9, and CD47 in PGCCs, exploring whether the ectopic expression of syncytin 1, CD9, and CD47 exists in PGCCs, compared to the case in control cells. In addition, various signaling cascades such as the cAMP/PKA and JNK pathways have been shown to mediate the syncytialization process.^16^, ^17^ Thus, we assessed the expressional differences of PKA RIα, JNK1, and c‐Jun between the control cells and PGCCs.

## 2 MATERIAL AND METHODS

### 2.1 Cell lines and cultures

The human colorectal cancer cell lines HCT116 and LoVo were obtained from the American Type Culture Collection (ATCC; USA). HCT116 and LoVo cells were cultured with RPMI Medium 1640 basic (1X) (Gibco, Thermo Fisher Scientific, Suzhou, China) supplemented with 10% fetal bovine serum (FBS; Gibco, Life technologies, New Zealand) and 1% penicillin streptomycin (PS; Gibco, Life technologies, USA). The appropriate temperature (37°C), CO_2_ (5%), and humidity conditions are also indispensable for the routine maintenance of the cells.

### 2.2 Formation of PGCCs

HCT116 and LoVo cells were regularly incubated in T25 flasks and cultured with complete 1640 medium. The cells were treated with an equal concentration (375 μM) of cobalt chloride (CoCl_2_; Sigma‐Aldrich, St. Louis, MO, USA) when they attained 60%‐70% confluence. The cells were cultured for different periods according to their specific hypoxia‐resistance capacities. HCT116 and LoVo cells were treated with 375 μM CoCl_2_ for 48‐72 hours and 30‐40 hours, respectively. Then, the cells were rinsed with phosphate‐buffered saline (PBS; Gibco, Thermo Fisher Scientific, Suzhou, China) and cultured with regular complete medium. A portion of regular‐sized cells died following treatment with CoCl_2_ and the cells that survived CoCl_2_treatment displayed morphological changes. Several days after removing CoCl_2_, PGCCs were observed; they started to produce daughter cells by budding. A sufficient number of PGCCs were acquired after 2 or 3 more CoCl_2_ treatments. The PGCCs (30%) and newly generated daughter cells (70%) were collected for further analysis.

### 2.3 Tumor tissue samples

The paraffin‐embedded tissue samples of human colorectal tumors (n = 173) were obtained from Tianjin People's Hospital (Tianjin, China). The patients were determined to suffer from colorectal cancer and none of them had received medical treatment for colorectal carcinoma before surgical resection. We divided these 173 cases of colorectal tumors into six groups: 52 cases of well‐differentiated colorectal cancer, including 47 cases of primary colorectal tumor (group I) and 5 cases of corresponding metastasis (group II); 52 cases of moderately differentiated colorectal tumor, including 38 cases of primary colorectal tumor (group III) and 14 cases of corresponding metastasis (group IV); and 69 cases of poorly differentiated colorectal carcinoma, including 42 cases of primary colorectal tumor (group V) and 27 cases of corresponding metastasis (group VI). The utilization of these tumor samples was permitted by the tissue bank of the Tianjin People's Hospital. Furthermore, the patient information has been kept strictly confidential.

### 2.4 Western blot analysis

The HCT116 and LoVo control cells without CoCl_2_treatment were collected when their confluence reached 80%. Likewise, the PGCCs and their generated daughter cells were collected when they attained a similar confluence. The cells were lysed on ice with 80‐150 μL of glacial radio‐immunoprecipitation assay (RIPA) lysis buffer (Roche, Germany) for 30 minutes, and then centrifuged at 14,000 rpm/min for 30 minutes at 4°C. The concentrations of the proteins were determined, and they were separated by 10% sodium dodecyl sulfate polyacrylamide gel (SDS‐PAGE). The protein bands were then transferred onto polyvinylidene fluoride (PVDF) membranes (GE, USA). The protein‐containing PVDF membranes were blocked with 5% defatted milk (BD, USA) in 1 × Tris‐buffered saline with 1% Tween‐20 (Sigma, USA) for 2 hours at room temperature. Then, the membranes were incubated with primary antibodies (Table S1) at 4°C for 14‐16 hours. The membranes were then incubated with secondary antibodies at room temperature for 2 hours. Protein expression was finally detected using the Chemidoc imaging system (BioRad, USA). The Image‐J software was used to analyze the gray value of each protein band after capturing images from the film processor. β‐actin was employed as a protein‐loading control, and all the western blot experimental results were repeated multiple times.

### 2.5 Immunocytochemical (ICC) staining

Control cells and PGCCs with daughter cells from the HCT116 and LoVo cell lines were cultured in complete medium until they reached a confluence of 80%. The HCT116 control cells and PGCCs with daughter cells were diluted 60‐ and 40‐fold, respectively; the LoVo control cells and PGCCs with daughter cells were diluted 40‐ and 30‐fold, respectively. After incubation for 48 hours, the cells were treated with ice‐cold methyl alcohol for 30 minutes and were then rinsed with phosphate‐buffered solution (PBS; Zhongshan Inc Beijing, China). The cells were then blocked with endogenous peroxidase inhibitor (Zhongshan Inc, Beijing, China) and subsequently, with goat serum (Zhongshan Inc Beijing, China) for 15 and 20 minutes, respectively, at room temperature. The primary antibodies (Table S1) were incubated overnight at 4°C for 16 hours. Prior to incubation with diaminobenzidine (DAB, Zhongshan Inc), the cells were treated with the secondary antibodies and horseradish peroxidase‐labeled streptomycin (Zhongshan Inc) for 20 and 15 minutes, respectively. The cells were then counterstained with hematoxylin. The cells were rinsed with twice with PBS and once with PBST before incubation with each of the reagents (except serum) for 5 minutes.

### 2.6 Immunohistochemical (IHC) staining

IHC staining was carried out for all the slices. Paraffin‐embedded tissue sections were subjected to hyperthermal roasting at 70°C for 2 hours, and were then deparaffinized in xylene and dehydrated using concentration‐gradient ethanol solutions. Next, antigen retrieval was performed using heated citrate buffer solution (Origene, Wuxi, China) in an autoclave at 100°C for 1.5 minutes. After blocking with endogenous peroxidase inhibitor and then with goat serum, the sections were incubated with the primary antibodies at 4°C for 16 hours. The further processes were the same as that post incubation with the primary antibodies in ICC staining.

### 2.7 Hematoxylin‐eosin (H&E) staining

H&E staining was performed for the slides with the control cells and PGCCs. The cells were irrigated with PBS twice for 5 minutes, and then steeped in ultrapure water for a few seconds. The slides were firstly dyed with hematoxylin (Baso, Zhuhai, China) for approximately 20 seconds before eosin‐hydrosoluble (Baso, Zhuhai, China) staining for 20‐30 seconds. Next, the cells were dehydrated with absolute ethyl alcohol for a few seconds. Finally, the mean numbers of PGCCs were counted by selecting 5 fields randomly.

### 2.8 IHC scoring

The expression of syncytin 1 in the tissue samples was evaluated and quantified based on the percentage of positive cells and staining intensity. Claybank staining in the cytoplasm was considered to be positive expression, and the percentage of positive cells was scored as follows: 0 (negative), <10% positive cells; 1 (weak), 11%‐30% positive cells; 2 (moderate), 31%‐70% positive cells, and 3 (strong), >70% positive cells. The staining intensity was stratified as follows: 0, negative (no staining); 1, weakly positive (pale yellow staining); 2, moderately positive (yellow staining); and 3, strongly positive (brown yellow staining). The product of the percentage of positive cells and staining intensity was responsible for the determination of staining index for each section.

### 2.9 Statistical analysis

The statistical software GraphPad prism 7 was used for all the statistical data analyses in this study. The Shapiro‐Wilk normality test was used to detect whether the data belongs to normal distribution or not. The Mann‐Whitney test was used to assess the differences between the expressions of cell fusion‐related proteins and cAMP/PKA and JNK signaling proteins among the different groups. The Kruskal‐Wallis test was used to compare the staining index differences of syncytin 1 among 3 groups. In this study, a *P*‐value <0.05 was considered significant.

## 3 RESULTS

### 3.1 Formation of PGCCs induced by CoCl_2_ in colorectal cancer cells

**Figure 1:**
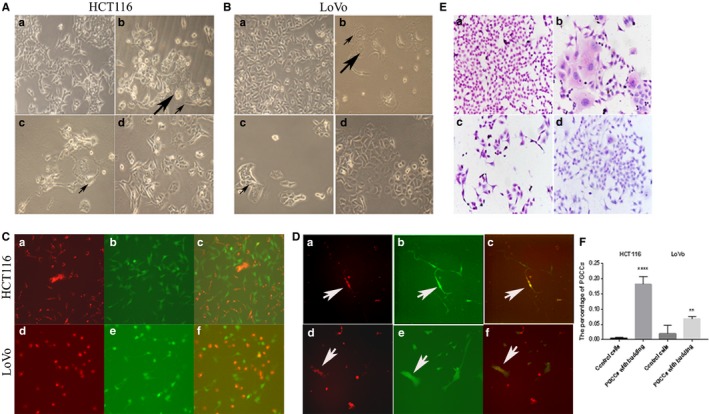
PGCCs with budding daughter cells. A, HCT116 PGCCs and control HCT116 cells. (a) Control HCT116 cells (100×). (b). HCT116 PGCCs induced by 375 μM CoCl_2_ treatment for 60 h (100×). (The large black arrow heads indicate the PGCCs; the small black arrow heads indicate the budding daughter cells; 200×). (c) The PGCCs generated daughter cells via budding. The black arrow heads indicate the budded daughter cells; 100×). (d) Generation of daughter cells by budding contributes to the reproduction of the PGCCs (100×). B, LoVo PGCCs and control LoVo cells. (a) Control LoVo cells (200×). (b) HCT116 PGCCs induced by 375 μM CoCl_2_ treatment for 30 h (100×). (The large black arrow heads indicate the PGCCs; the small black arrow heads indicate the budding daughter cells; 100×). (c) The PGCCs generated daughter cells via budding. The black arrow heads indicate the budded daughter cells; 100×). (d) Generation of daughter cells by budding contributes to the reproduction of the PGCCs (100×). C, Fluorescent markers of different colors were used to detect the cell fusion in HCT116 and LoVo before CoCl_2_ treatment (100×). (a) HCT116 cells with red fluorescence before CoCl_2_ treatment. (b) HCT116 cells with green fluorescence before CoCl_2_ treatment. (c) Merge image of (a) and (b). (d) LoVo cells with red fluorescence before CoCl_2_ treatment. (e) LoVo cells with green fluorescence before CoCl_2_ treatment. (f) Merge image of (d) and (e). D, Fluorescent markers of different colors were used to detect the cell fusion in HCT116 and LoVo after CoCl_2_ treatment (100×). (a) HCT116 cells with red fluorescence after CoCl_2_ treatment and white arrow points the PGCC. (b) HCT116 cells with green fluorescence after CoCl_2_ treatment and white arrow points the same PGCC of (a). (c) Merge image of (a) and (b) and white arrow points the PGCC with yellow. (d) LoVo cells with red fluorescence after CoCl_2_ treatment and white arrow points the PGCC. (e) LoVo cells with green fluorescence after CoCl_2_ treatment and white arrow points the same PGCC of (d). (f) Merge image of (d) and (e) and white arrow points the PGCC with yellow. E, H&E staining of the HCT116 and LoVo cells before and after CoCl_2_ treatment (100×). (a) H&E staining of the control HCT116 cells. (b) Many PGCCs appeared in HCT116 cells after CoCl_2_ treatment. (c) H&E staining of the control LoVo cells. (d) Many PGCCs appeared in LoVo cells after CoCl_2_ treatment. F, Quantitative results of the percentage of PGCCs of control cells and PGCCs of HCT116 and LoVo cells

When cells from the colorectal cancer cell lines HCT116 and LoVo were treated with a low concentration of CoCl_2_, a part of regular‐sized diploid tumor cells were killed, while several sporadic large cells with multiple or giant nuclei (PGCCs) and cancer cells with altered morphology survived. In comparison to the HCT116 control cells (1A‐a), a part of the diploid cancer cells were killed and surviving PGCCs were observed following the treatment of the HCT116 cells with a low concentration of CoCl_2_ (375 μM) for 60 hours (Figure 1A (b)). Analogical morphological changes occurred and were observed in the LoVo colorectal cancer cell line (Figure 1B (a,b)). Two weeks after the removal of CoCl_2_and dead cells, the surviving PGCCs with budding cells could be observed in HCT116 (Figure 1A (c)) and LoVo (Figure 1B (c)) and the separate percentages of the PGCCs and daughter cells, were 40% and 60%, respectively. When the cells reached a confluence of 80%, during which the number of PGCCs and their daughter cells accounted for 30% and 70%, respectively, along with an increasing number of budding cells in HCT116 (Figure 1A (d)) and LoVo (Figure 1B (d)), these PGCCs with daughter cells and the control cells were collected to analyze the expression of cell‐cell fusion‐related proteins.

To assess whether cell fusion promote the formation of PGCCs, fluorescence of different colors was marked in HCT116 and LoVo cells (Figure 1C and D). We labeled HCT116 and LoVo control cells with red and green fluorescence, respectively, and then equivalent red cells and green cells were mixed and cultured in medium. Without CoCl_2_ treatment, the mixed cells appeared either red (Figure 1C (a) (HCT116) and Figure 1C (d) (LoVo)) or green fluorescence (Figure 1C (b) (HCT116) and Figure 1C (e) (LoVo)) merely, and no yellow cells appeared in the merge phase (Figure 1C (c) (HCT116) and Figure 1C (f) (LoVo)). While the mixed cells were treated with CoCl_2_, some survived PGCCs appeared both red (Figure 1D (a) (HCT116) and Figure 1D (d) (LoVo)) and green (Figure 1D (b) (HCT116) and Figure 1D (e) (LoVo)) and these PGCCs were yellow in the merge phase (Figure 1D (c) (HCT116) and Figure 1D (f) (LoVo)). All these data showed that PGCCs formation could be induced via cell fusion in response to CoCl_2_.

To further explore whether the number of PGCCs is different between control cell cultures and cell cultures after CoCl_2_treatment, H&E staining was performed on the HCT116 and LoVo control cells and PGCCs (Figure 1E). Then, the numbers of PGCCs in the different groups were counted. The results show that the number of PGCCs is lesser in control cell cultures than in cell cultures after CoCl_2_ treatment in case of both HCT116 (Figure 1E (a,b), *t*=−15.405, Table 1) and LoVo (Figure 1E (c,d), *t*=−3.991, Table 1) cells. These data were statistically significant for HCT116 (*P* = 0.000, Figure 1F) and LoVo (*P* = 0.004, Figure 1F) cells.

**Table 1:** The percentage of PGCCs in HCT116 and LoVo cells before and after CoCl_2_ treatment

HCT116	Percentage of PGCCs	*t*	*P*	LoVo	Percentage of PGCCs	*t*	*P*
Control	0.01 ± 0.00	−15.405	0.000	Control	0.02 ± 0.03	−3.991	0.004
CoCl_2_	0.18 ± 0.03			CoCl_2_	0.07 ± 0.01		

### 3.2 Fusion‐related protein expression in control colorectal cancer cells and PGCCs

**Figure 2:**
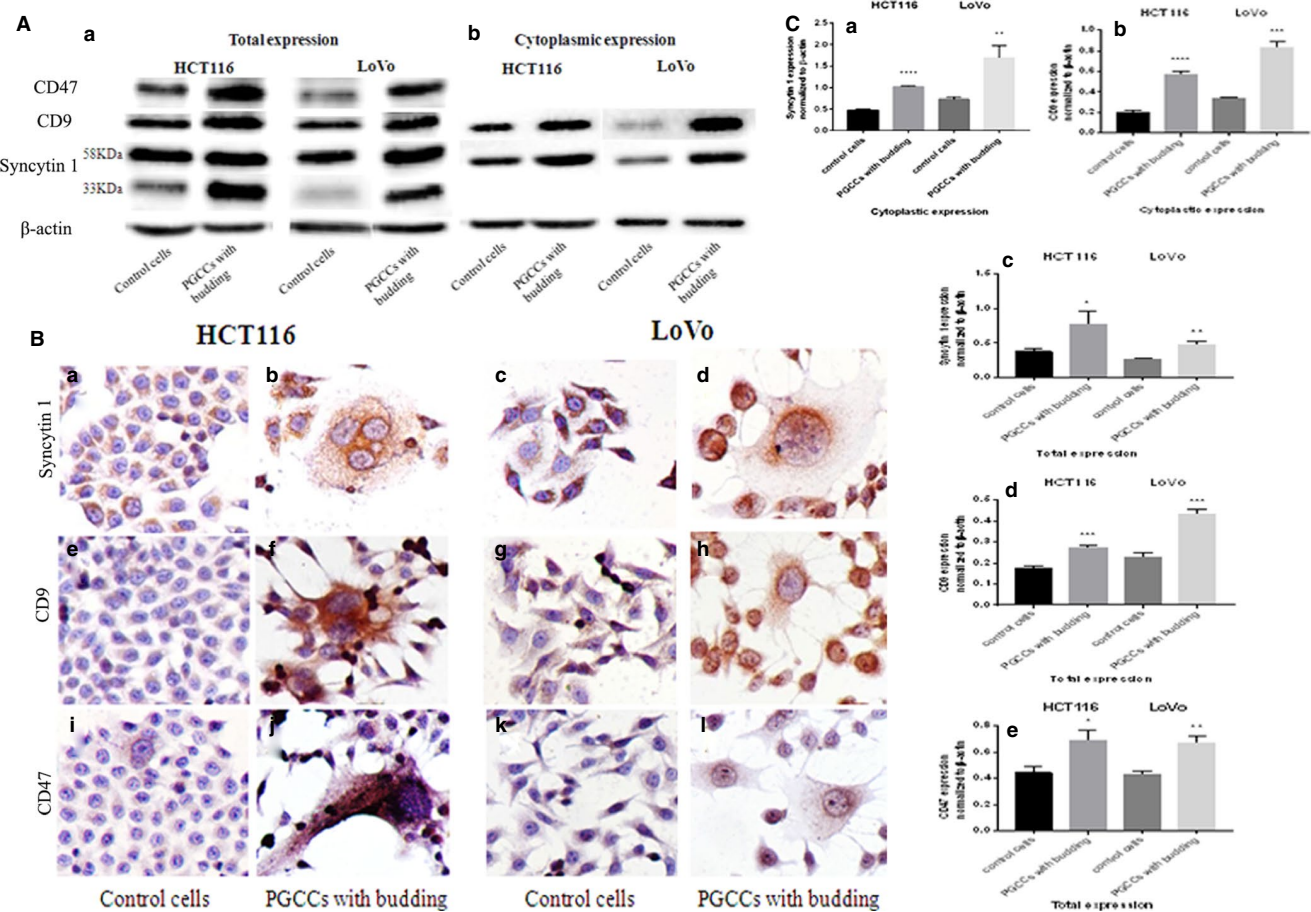
Syncytin 1, CD9, and CD47 expression in HCT116 and LoVo PGCCs with budding and HCT116 and LoVo control cells. A, Western blotting was used to assess differences in syncytin 1, CD9, and CD47 expression in HCT116 and LoVo cells before and after CoCl_2_ treatment. (a) Total expression of syncytin 1, CD9, and CD47 in the HCT116 and LoVo control HCT116 and LoVo cells and HCT116 and LoVo PGCCs. (b) Cytoplasmic expression of syncytin 1 and CD9 in the control HCT116 and LoVo cells and HCT116 and LoVo PGCCs. B, ICC staining was used to detect the subcellular location of syncytin 1, CD9, and CD47 in HCT116 and LoVo PGCCs with budding and control HCT116 and LoVo cells. (a) The subcellular location of syncytin 1 in control HCT116 and LoVo cells and HCT116 and LoVo PGCCs. (b) The subcellular location of CD9 in control HCT116 and LoVo cells and HCT116 and LoVo PGCCs in. (c) The subcellular location of CD47 in control HCT116 and LoVo cells and HCT116 and LoVo PGCCs. C, Quantitative results of total and cytoplasmic protein expression differences are shown as histograms. (a) The histogram of cytoplasmic syncytin 1 expression in HCT116 and LoVo. (b) The histogram of cytoplasmic CD9 expression in HCT116 and LoVo. (c) The histogram of total syncytin 1 expression in HCT116 and LoVo. (d) The histogram of total CD9 expression in HCT116 and LoVo. (e) The histogram of total CD47 expression in HCT116 and LoVo. The corresponding densitometric analyses of each protein band were performed using image‐J software; the signals of each protein band were normalized to the β‐actin signal

To investigate whether the formation of PGCCs was correlated with cell fusion, the expression levels and subcellular locations of fusion‐related proteins including syncytin 1, CD9, and CD47 were analyzed in control HCT116 and LoVo cells and in PGCCs with daughter cells. The western blot analysis indicates that the total expression of syncytin 1, CD9, and CD47 was low in control cells; however, they were highly expressed in PGCCs and the cells generated by these PGCCs (Figure 2A (a)), indicating that the formation of PGCCs may be dependent on cell fusion. Western blotting analysis for the isolation of the cytoplasmic and nuclear proteins syncytin 1, CD9, and CD47 was also carried out. The cytoplasmic expression of syncytin 1 and CD9 in PGCCs was higher than that in control cells (Figure 2A (b)), while nuclear syncytin 1 and CD9 were not detected in both control cells and PGCCs. However, the CD47 protein was detected neither in the cytoplasm nor in the nucleus in both control cells and PGCCs (data not shown); this could have possibly resulted from the loss of CD47 during the separation process of cytoplasmic proteins from nucleoproteins. Notably, the molecular weights of syncytin 1 were found to be 33 and 58 KDa before the isolation of the cytoplasmic syncytin 1 from the nuclear syncytin 1. There was no difference between the expressions of syncytin 1 with a molecular weight of 58 KDa in the control cells and PGCCs, and the expression of syncytin 1 with a molecular weight of 33 KDa was higher in PGCCs than in the control cells. However, the molecular weight of cytoplasmic syncytin 1 was 58 KDa (Figure 2A (a,b)).

We also evaluated the subcellular location of syncytin 1, CD9, and CD47 with ICC staining, showing that these 3 fusion‐related proteins were expressed mainly in the cytoplasm in control cells and PGCCs (Figure 2B), consistent with the western blotting results (for both HCT116 and LoVo cells). Interestingly, CD9 and CD47 were uniformly distributed in the cytoplasm of PGCCs and daughter cells (Figure 2B‐F (h,j,i), while syncytin 1 mainly gathered around the nucleus and showed a gradual declining tendency from the cell nucleus to the margin of the cytoplasm in PGCCs (Figure 2B (b,d)), which led to our assumption that syncytin 1 might be involved in the fusion of regular‐sized cell nuclei during the formation of PGCCs (for both HCT116 and LoVo cells). Moreover, the ICC staining results also displayed a visually and significantly increased expression of syncytin 1 and CD9, and a relatively slight increase of CD47 expression within the cytoplasm of PGCCs (Figure 2,d,f,h,j,l)), compared to the case in control cells (Figure 2,c,e,g,i,k)), which was consistent with the western blotting results (for both HCT116 and LoVo cells). Interestingly, we also observed that the expression of CD47 within PGCCs, which was visible occasionally in HCT116 control cells, was higher than that in the surrounding control cells (Figure 2B (i)), corresponding with the fact that the PGCC formation was induced by CoCl_2_, and showing that the increased expression of fusion‐related proteins may be a common phenomenon in PGCCs. A quantitative analysis of fusion‐related proteins syncytin 1, CD9, and CD47 expression in control cells and PGCCs was performed; it showed a remarkable difference between the control cells and PGCCs (Figure 2C). Notably, the densitometric analyses of all protein bands were standardized with the corresponding beta‐actin bands.

### 3.3 Expression of PKA RIα, JNK1, and c‐Jun in control colorectal cancer cells and PGCCs

**Figure 3:**
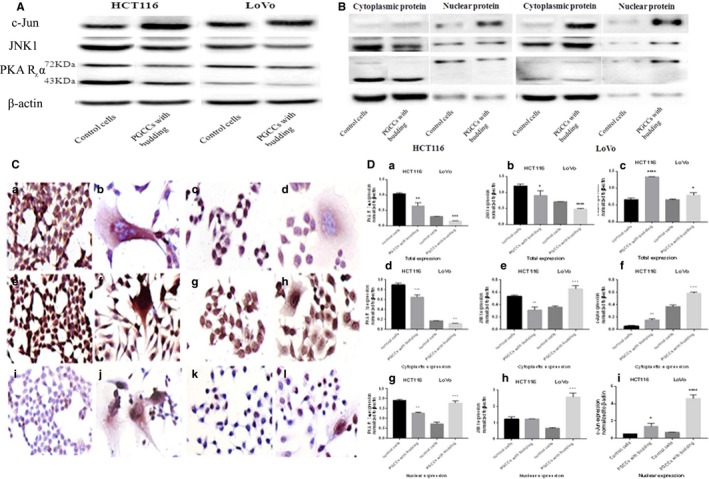
PKA RIα, JNK1, and c‐Jun expression in HCT116 and LoVo PGCCs with budding and control cells. A, Western blotting was used to test differences in the total expression of PKA RIα, JNK1, and c‐Jun in HCT116 and LoVo cells before and after CoCl_2_ treatment. B, Cytoplasmic and nuclear expression of PKA RIα, JNK1, and c‐Jun in control HCT116 and LoVo cells and HCT116 and LoVo PGCCs. C, ICC staining was used to assess the subcellular location of PKA RIα, JNK1, and c‐Jun in HCT116 and LoVo PGCCs with budding and control cells (100×). (a) PKA RIα ICC staining in HCT116 control cells. (b) PKA RIα ICC staining in HCT116 PGCCs. (c) PKA RIα ICC staining in LoVo control cells. (d) PKA RIα ICC staining in LoVo PGCCs. (e) JNK1 ICC staining in HCT116 control cells. (f) JNK1 ICC staining in HCT116 PGCCs. (g) JNK1 ICC staining in LoVo control cells. (h) JNK1 ICC staining in LoVo PGCCs. (i) c‐Jun ICC staining in HCT116 control cells. (j) c‐Jun ICC staining in HCT116 PGCCs. (k) c‐Jun ICC staining in LoVo control cells. (l) c‐Jun ICC staining in LoVo PGCCs. D, Quantitative results of total, cytoplasmic, and nuclear protein expression differences are shown as histograms. The corresponding densitometric analyses of each protein band were performed using image‐J software; the signals of each protein band were normalized to the β‐actin signal. (a) The histogram of total PKA RIα expression in HCT116 and LoVo. (b) The histogram of total JNK1 expression in HCT116 and LoVo. (c) The histogram of total c‐Jun expression in HCT116 and LoVo. (d) The histogram of cytoplasmic PKA RIα expression in HCT116 and LoVo. (e) The histogram of cytoplasmic JNK1 expression in HCT116 and LoVo. (f) The histogram of cytoplasmic c‐Jun expression in HCT116 and LoVo. (g) The histogram of nuclear PKA RIα expression in HCT116 and LoVo. (h) The histogram of nuclear JNK1 expression in HCT116 and LoVo. (i) The histogram of nuclear c‐Jun expression in HCT116 and LoVo

Western blot analysis was performed for the PKA RIα, JNK1, and c‐Jun proteins, and their total, cytoplasmic, and nuclear protein expression tendency, was evaluated in control cells and PGCCs separately. The molecular weights of PKA RIα were about 43 and 72 KDa and their total expression displayed a similar reduced trend in PGGCs compared to control cells (HCT116 and LoVo) (Figure 3A). The expression of JNK1 decreased in PGCCs (HCT116 and LoVo) (Figure 3A). Moreover, c‐Jun displayed an increased expression trend in PGGCs compared to control cells (HCT116 and LoVo) (Figure 3A). In the analysis of the differential expression between cytoplasmic and nuclear proteins, we observed that both the cytoplasmic and nuclear expression of PKA RIα showed a decreased tendency in HCT116 PGCCs, while it displayed an increased trend in LoVo PGCCs. Furthermore, we observed that the cytoplasmic PKA RIα showed molecular weights of 43 and 72 KDa, while the nuclear PKA RIα only showed a molecular weight of 72 KDa (Figure 3B). Thus, we supposed that the nuclear PKA RIα was subjected to a modification. In HCT116 cells, the cytoplasmic JNK1 expression was lower in PGCCs than control cells. There was no difference between the control cells and PGCCs for the nuclear JNK1 expression. For LoVo cells, the expression of cytoplasmic and nuclear JNK1 displayed a slightly increased trend in PGCCs compared with the control cells (Figure 3B). Moreover, the expression of cytoplasmic and nuclear c‐Jun was greater in PGCCs than in control cells in case of both the HCT116 and LoVo cells (Figure 3B).

To further investigate whether the subcellular locations of PKA RIα, JNK1, and c‐Jun were different after treatment with a low concentration of CoCl_2_, ICC staining was performed in HCT116 and LoVo cells. We found that PKA RIα was expressed in the cytoplasm and nucleus in control cells and that cytoplasmic PKA RIα was highly expressed in PGCCs, while it was poorly expressed in the daughter cells (HCT116 and LoVo) (Figure 3C). Analysis for JNK1 demonstrated that it was detected both in the cytoplasm and nucleus in cells without CoCl_2_treatment. However, JNK1 was expressed in the cytoplasm in all PGCCs and accompanied by its segmental transportation to the nucleus (Figure 3C). In addition, c‐Jun was barely observed in control cells, compared with its evident transportation into the nucleus in all PGCCs and partial daughter cells (in case of both HCT116 and LoVo cells) (Figure 3C).

The quantitative analysis for PKA RIα, JNK1, and c‐Jun expression in control cells and PGCCs illustrates a significant difference between the control cells and PGCCs (Figure 3D). Additionally, the densitometric analyses of all protein bands were standardized with the corresponding β‐actin bands.

### 3.4 Expression of syncytin 1 in human colorectal tumor tissues

**Figure 4:**
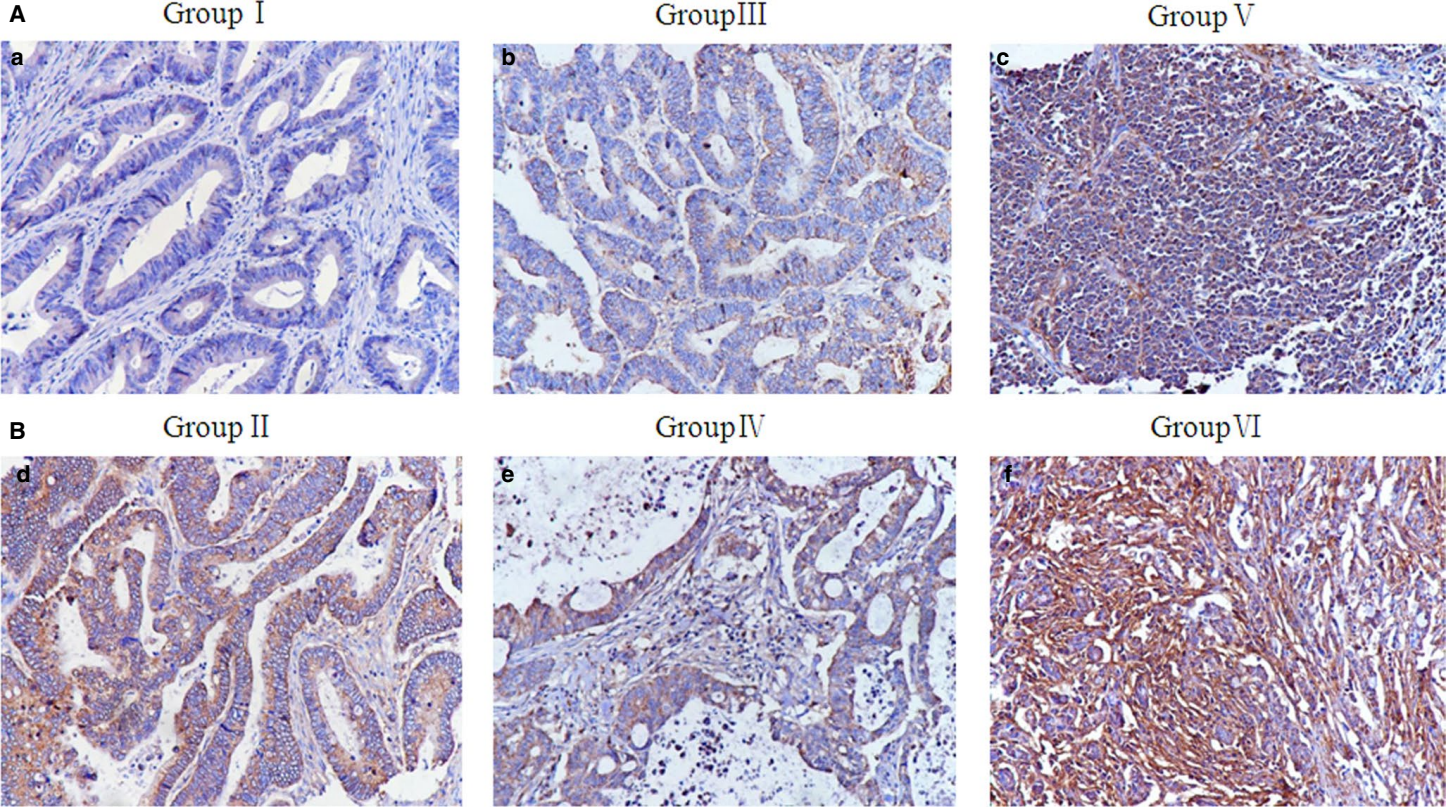
The expression of syncytin 1 in human colorectal tumor tissues. A, Syncytin 1 expression in (a) well‐differentiated primary colorectal cancer (group I), (b) moderately differentiated primary colorectal cancer (group II), and (c) poorly differentiated primary colorectal cancer (group III) (200×). B, Corresponding metastases. (d) Corresponding metastasis of the well‐differentiated primary colorectal cancer (group IV), (e) Corresponding metastasis of the moderately differentiated primary colorectal cancer (group V), and (f) Corresponding metastasis of the poorly differentiated primary colorectal cancer (group VI) (200×)

To test the syncytin 1 expression level and its clinicopathological significance, IHC staining for syncytin 1 was carried out on 173 samples of formalin‐fixed and paraffin‐embedded human colorectal tumor tissues. A positive syncytin 1 staining index was detected in the cytoplasm of tumor cells among the 6 groups. The syncytin 1 expression was higher in case of well‐differentiated cancer with metastasis (group II) than in the corresponding primary tumors (group I) (Figure 4 B (d) and A (a), Table 2), higher in case of moderately differentiated cancer with metastasis (group IV) than in the corresponding primary tumors (group III) (Figure 4 B (e) and A (b), Table 2), higher in case of poorly differentiated cancer with metastasis (group VI) than in the corresponding primary tumors (group V) (Figure 4 B (f) and A (c), Table 2), higher in case of moderately differentiated primary cancer (group III) than in the well‐differentiated primary cancer (group I) (Figure 4b) 4nd A (a), Table 2), and higher in case of poorly differentiated primary cancer (group V) than in moderately differentiated primary cancer (group III) (Figure 4) 4nd A (b), Table 2). Statistical analysis suggested that the syncytin 1 expression was lower in group I than in group II (*Z*=−3.829, *P* = 0.000, Table 2), lower in group III than in group IV (*Z*=−9.101, *P* = 0.000, Table 2) and lower in group V than in group VI (*Z*=−2.549, *P* = 0.029, Table 2), lower in group I than in group III (*Z*=−6.121, *P* = 0.001, Table 2), and lower in group III than in group V (*Z*=−6.130, *P* = 0.000, Table 2). Additionally, there were significant differences in the syncytin 1 expression between groups I, III, and V (χ^2^ =0.961, *P* = 0.000, Table 3).

**Table 2:** The differences of syncytin 1 expression between group I and group II, group III and group IV, group V and group VI

	Group	Number	Syncytin 1	*Z*	*P*
Well‐differentiated tumor	I	47	1.60 ± 1.25	−3.829	0.000
Metastasis of well‐Differentiated tumor	II	5	5.60 ± 2.30		
Moderately differentiated tumor	III	38	2.92 ± 1.78	−9.101	0.000
Metastasis of moderately differentiated tumor	IV	14	5.00 ± 1.24		
Poorly differentiated tumor	V	42	6.02 ± 2.69	−2.549	0.029
Metastasis of poorly differentiated tumor	VI	27	7.44 ± 1.93		
Well‐differentiated tumor	I	47	1.60 ± 1.25	−6.121	0.001
Moderately differentiated tumor	III	38	2.92 ± 1.78		
Moderately differentiated tumor	III	38	2.92 ± 1.78	−6.130	0.000
Poorly differentiated tumor	V	42	6.02 ± 2.69		

**Table 3:** The differences of syncytin 1 expression between group I, III, and V

	Group	Number	Syncytin 1	χ^2^	*P*
Well‐differentiated tumor	I	47	1.60 ± 1.25	0.961	0.000
Moderately differentiated tumor	III	38	2.92 ± 1.78		
Poorly differentiated tumor	V	42	6.02 ± 2.69		

## 4 DISCUSSION

PGCCs were initially observed after treatment with a high concentration of CoCl_2_ and the regular diploid cells were selectively killed.^18^ PGCCs are large and contribute to solid tumor heterogeneity. The shape of the nucleus of PGCCs is usually irregular and PGCC nuclei are at least 3 to 5 times larger in size than regular‐sized diploid cancer cell nuclei.^19^ The PGCCs can be induced by CoCl_2_through endoreduplication or cell fusion and revert to regular cancer cells via budding, splitting, or burst‐like mechanisms commonly observed in simple organisms. Overexpression of the cell cycle regulatory proteins Cyclin E, SKP2, Stathmin, p38, phosphorylated kinase 1, protein kinase C, phosphorylated AKT, and mitogen‐activated protein kinase (MAPK) has been detected in PGCCs, but not in diploid cells.^20^ The results of our recent study elucidated that the ectopic expression and subcellular location of the cell cycle‐related proteins Cyclin B1 and CDC25 play an important role in formation of PGCCs. In this paper, we analyzed the expression and subcellular location of fusion‐related proteins including syncytin 1, CD9, and CD47, and other proteins that may be involved in cell fusion, namely, PKA RIα in the cAMP/PKA pathway, and JNK1 and c‐Jun in the JNK pathway.

Syncytin 1 mainly facilitates the formation of syncytium in various types of cells. The results of our study indicate that the expression of syncytin 1 was significantly increased in PGCCs and their daughter cells, showing that the formation of PGCCs may be attributed to cell fusion. Previous reports have suggested that a polypeptide comprising 538 amino acids encoded by the ERVWE1 gene is posttranslationally cleaved into the surface and transmembrane subunits and that the cytoplasmic domain of syncytin 1 regulates its fusogenic activity.^21^ Our results showed that the upregulation of cytoplasmic syncytin 1 with a molecular weight of 58 KDa might promote the formation of PGCCs through cell fusion. Interestingly, we observed that syncytin 1 clustered around the nucleus of PGCCs and gradually reduced from the nucleus to the cell edges. It might be possible that the ectopic expression of syncytin 1 has a positive role for nucleus fusion during PGCC formation. Furthermore, the daughter cells with high expression of syncytin 1 might be able to form PGCCs through fusion.

Herein, we demonstrated that the expression of CD9 was remarkably increased in PGCCs compared to the control cells. In sperm‐oocyte fusion, a possibility would be that the fusion process might be a direct consequence of CD9‐controlled adhesion, because an increased expression of CD9 has been reported to result in enhanced adhesion ability, and adhesion is the first and most essential step in any fusion process.^13^, ^22^ Moreover, the spatial distribution of CD9 plays an important role in, and might be a prerequisite for membrane adhesion and subsequent fusion events.^8^ Therefore, it was attractive to speculate that the spatial distribution of CD9 is possibly correlated with CD9‐induced generation of adhesion sites; the adhesion sites induced by CD9 may well be the actual locations where fusion occurs.^22^ Similarly, the forced expression of CD9 might promote the formation of PGCCs, based on elevated adhesion ability and subsequent fusion.

CD47 has been previously shown to take part in the formation of osteoclast and macrophage multinucleation through cell fusion. Other experiments have revealed that CD47 required a binding partner or delivered another signal to regulate cell fusion.^14^ CD47 was possibly involved in regulating the process of macrophage multinucleation as a ligand via attachment and fusion.^23^ Another possibility might be that CD47 may create pores to trigger cell‐cell fusion when the membranes are close to each other.^9^ Our results clearly indicate that CD47 expression increased after treatment with CoCl_2_. It is likely to act as a ligand or deliver signals to mediate cell fusion events during PGCC formation.

PKA, also known as cAMP‐dependent kinase, is one of the most common protein kinases. PKA exists as a heterotetramer that consists of 2 regulatory (R) and two catalytic (C) subunits. When cAMP molecules bind to R subunits in the holoenzyme, catalytically active C subunits are released, followed by a conformational change.^24^ Previously, it has been shown that the PKA pathway mediates cell fusion events by regulating the syncytin expression as an upstream signal and that the overexpression of an active C subunit was sufficient to increase syncytin expression and cell fusion.^25^ Furthermore, a report has proved that the PKAR1 and PKAR2 isoforms in *Mucor circinelloides* were posttranslationally modified by ubiquitylation. This modification has been identified to regulate the cAMP‐binding capacity of the R subunits PKAR1 and PKAR2, thereby regulating the holoenzyme kinase activity.^26^ In our study, we observed that the expression of PKA RIα in PGCCs with daughter cells displayed a weaker diminution than control cells. For this phenomenon, we proposed a hypothesis that the treatment of CoCl_2_, to some extent, promotes the degradation of ubiquitination‐modified PKA RIα, and the release of the C subunit. Jun N‐terminal Kinase (JNK) signaling could regulate the response to cell stress through cell death, proliferation, and migration. Cell fusion can also be promoted by the activation of the JNK pathway 27; however, the specific molecular mechanisms are not yet clear. In wound healing,^28^ previous findings have revealed that the expression of JNK was relatively high, and that JNK functions as a positive signal to regulate the cell fusion process. In addition, when JNK signaling is activated, the balance between JNK and JAK/STAT signaling may be a crucial determinant for fusion events. Interestingly, wound‐induced cell fusion was not found to be suppressed after JNK loss. Our results showed that the expression of JNK reduced slightly and the expression of c‐Jun increased remarkably. During wound healing, JNK activation was prominent 4 hours after injury, peaked at approximately 8 hours, and then gradually decreased. Whether the expression level of JNK has a similar trend with regards to time is unclear. c‐Jun is activated by phosphorylation, mediated by JNK, and then functions through its translocation into the nucleus. Accordingly, we speculate that c‐Jun upregulation may be involved in cell fusion through JNK signaling.

Previous data documented that the number of PGCCs was associated with the invasion and metastasis of malignant solid tumors.^29^ Zhang et al 18 indicated that the number of PGCCs increased dramatically with increased stage and tumor grade. Our results suggest that the expression of syncytin 1 associated with the grade and metastasis. We speculate that the overexpression of the fusogenic protein syncytin 1 may contribute to tumor metastasis by promoting cell fusion and PGCC formation.

Our data suggest that these fusion‐related proteins and cAMP/PKA and JNK signaling may represent useful fusogenic indicators for formation of PGCCs. The current study may serve as rationale for further investigation of the role of proteins syncytin 1, CD9, CD47 and signaling PKA RIα, JNK1 and c‐Jun in formation of PGCCs.

## CONFLICT OF INTEREST

No potential conflict of interest was disclosed.

## AUTHOR CONTRIBUTIONS

SZ designed the study; collected, analyzed, and interpreted data; contributed to manuscript writing; and approved the manuscript before submission. LC and FF collected and analyzed data and approved the manuscript before submission. XW, JD and KL collected, analyzed, and interpreted data, contributed to manuscript writing, and approved the manuscript before submission. BL, PY and YL collected data, gave constructive comments on the manuscript, and approved the manuscript before submission.
