# Supplementary material for: Syncytin 1, CD9, and CD47 regulating cell fusion to form PGCCs associated with cAMP/PKA and JNK signaling pathway
Source: Cancer Med. 2019 Apr 25;8(6):3047–58. doi: 10.1002/cam4.2173 (PMC6558479; doi:10.1002/cam4.2173)
Supplement: Supplementary file 1 [file CAM4-8-3047-s001.doc]

**Supplementary table 1** Detail information of antibodies used in western blot (WB) and immunocytochemical (ICC) staining

| Antibodies | Companys | Dilution (WB/ICC) | Dilution (WB/ICC) |
| --- | --- | --- | --- |
|  |  | HCT116 | LoVo |
| Syncytin 1 | Bioss | 1:1200/1:1200 | 1:1200/1:1200 |
| CD9 | Bioss | 1:1200/1:1000 | 1:1200/1:1000 |
| CD47 | Bioss | 1:1200/1:600 | 1:1200/1:600 |
| PKA RⅠα | abcam | 1:5000/1:1000 | 1:5000/1:300 |
| JNK1 | Proteintech | 1:3000/1:20000 | 1:3000/1:8000 |
| c-Jun | CST | 1:1000/1:500 | 1:1000/1:1000 |
